# Enteral immunonutrition versus enteral nutrition for gastric cancer patients undergoing a total gastrectomy: a systematic review and meta-analysis

**DOI:** 10.1186/s12876-018-0741-y

**Published:** 2018-01-16

**Authors:** Ying Cheng, Junfeng Zhang, Liwei Zhang, Juan Wu, Zhen Zhan

**Affiliations:** School of medicine and life sciences, Nanjing University of Chinese Medicine, 138 Xianlin Rd, Nanjing, China

**Keywords:** Enteral immunonutrition, Enteral nutrition, Gastrectomy, Gastric cancer

## Abstract

**Background:**

Nutrition support is a common means for patients with gastric cancer, especially for those undergoing elective surgery. Recently, enteral immunonutrition (EIN) was increasingly found to be more effective than enteral nutrition (EN) in enhancing the host immunity and eventually improving the prognosis of gastric cancer patients undergoing gastrectomy. However, the results reported were not consistent. This meta-analysis aimed to assess the impact of EIN for patients with GC on biochemical, immune indices and clinical outcomes.

**Methods:**

Four electronical databases (Medline, EMBASE, Scopus and Cochrane library) were used to search articles in peer-reviewed, English-language journals. Mean difference (MD), Relative risk (RR), or standard mean difference (SMD) with 95% confidence interval (CI) were calculated. Heterogeneity was assessed by Cochrane Q and I^2^ statistic combined with corresponding *P*-value. The analysis was carried out with RevMan 5.3.

**Results:**

Seven studies involving 583 patients were eligible for the pooled analysis. EIN, when beyond a 7-day time-frame post-operatively (D ≥ 7), increased level of CD4^+^ (SMD = 0.99; 95% CI, 0.65–1.33; *P* < 0.00001), CD4^+^/ CD8^+^ (SMD = 0.34; 95% CI, 0.02–0.67; *P* = 0.04), the IgM (SMD = 1.15; 95% CI, 0.11–2.20; *P* = 0.03), the IgG (SMD = 0.98; 95% CI, 0.55–1.42; *P* < 0.0001), the lymphocyte (SMD = 0.69; 95% CI, 0.32–1.06; *P* = 0.0003), and the proalbumin (SMD = 0.73; 95% CI, 0.33–1.14; *P* = 0.0004). However, those increased effects were not obvious within a 7-day time-frame post-operatively (D < 7). The levels of CD8^+^ and other serum proteins except proalbumin were not improved both on D ≥ 7 and D < 7. Clinical outcomes such as systemic inflammatory response syndrone (SIRS) (MD, - 0.89 days; 95% CI, - 1.40 to - 0.39; *P* = 0.005), and postoperative complications (RR, 0.29; 95% CI, 0.14–0.60; *P* = 0.001) were significantly reduced in EIN group. Pulmonary infection and length of hospitalization (LHS) were not improved no matter what time after surgery.

**Conclusions:**

EIN was found to improve the cellular immunity, modulate inflammatory reaction and reduce postoperative complication for GC patients undergoing radical gastrointestinal surgery. Exclusion of grey literature and non-English language studies was the key limitation in this study.

## Background

As a common digestive system tumor, patients with gastric cancer (GC) are often prone to malnutrition, and it might worsen by elective surgery [1, 2]. Malnutrition represents a factor, which was associated with immune function depression, inflammation response alteration, and exaggeration of stress response. Thus, these patients often have poor outcome of surgery in several aspects, such as infectious complications, wound healing delay or failure and a consequent longer hospital stay [3].

From nutritional point of view, supplements of nutrition by means of parenteral or enteral feeding, has been proposed to be an essential adjuvant therapy of surgical patients. The choice of enteral nutrition (EN) or parenteral nutrition (PN) depends on each patient’s gut function and tolerance of nutrient supply patterns [4]. EN following major gastrointestinal surgery is recommended over PN in surgical wards due to more in line with physiological characteristics and lower complications and costs, when the patient’s intestinal function allows the case. Although essential energy, protein, fat, carbohydrate, mineral, vitamin etc. were provided, the effect of EN was less significant than expected [5]. Recently, enteral immunonutrition (EIN) including ω-3 fatty acids, glutamine (Gln), arginine (Arg), and nucleotide has received increasing attention [6].

EIN has been reported to be an important treatment to reduce postoperative infection and noninfectious complications, raise the host immunity, and ameliorate the prognosis of patients suffering from gastrointestinal cancer [7, 8]. For instance, Arg is a semiessential amino acid with multiple roles in cellular metabolism [9]; Gln is a necessary nutrient for intestinal mucosal cell metabolism. In the severe stress, such as surgery, infection, the intestinal mucosal epithelial cells of glutamine are depleted rapidly resulting in impaired intestinal immune function [10]. In addition, other immune-nutrition, such as ω-3-FAs also has immunomodulatory and anti-inflammatory properties.

Although the effect of EIN on clinical outcome, immunological level, nutrition status was compelling, not all researches demonstrated similar clinical benefits and some studies have contradictive results [6]. The inconsistency of the results may due to heterogeneity among studies (i.e. different disease type and demographic characteristics, inclusion of parenteral nutrition, nutritional or metabolic status and time).

Zhang et al. in 2012 conducted a systematic review regarding immunonutrition vs standard diet in gastrointestinal cancer patients, however, only length of hospital stay and morbidity of infectious complication after surgery was calculated [11]. Recently, Wong et al. also reported a clinical beneficial effect of EIN vs EN in decreasing wound infection rate and reduction of hospital stay in upper gastrointestinal surgery [12]. However, mixture of all digestive system malignancies (whatever upper and lower gastrointestinal surgery) may results in heterogeneity and limited application. For GC patients, the pooled results have been reported by a meta-analysis [13, 14], however, the search terms about “EIN” used only was “enteral immunonutrition” with medical subject heading. Two studies with specific immunonutrition elements were not included. Herein, we conducted an update meta-analysis to comprehensively assess the effect of EIN compared with EN for GC patients regarding both laboratory indices and clinical outcomes.

## Methods

### Retrieval strategy

Medline (PubMed, 1966 to October 31, 2016), EMBASE (OVID, 1980 to October 31, 2016), Scopus (1995 to October 31, 2016) and Cochrane library were used. Medical subject heading (MeSH) and Thesaurus were used in PubMed and OVID, respectively. According the PICOs, the keywords were determined and identical in the two database (Medline and EMBASE): “Neoplasms”, “Gastric Neoplasm”, “Gastric Cancer”, “Gastric Tumor”, “Gastric Carcinoma”, “Stomach Neoplasms”, “Stomach Cancer”, “Stomach Carcinoma”, “gastrointestinal tract”, “Arginine”, “Glutamine”, “ω-3 Fatty Acids”, “Nutritional Support”, “Enteral Immune Nutrition”, “Nutrition”, “Immune-Enhancing Enteral Nutrition”, “Immunoenhanced Enteral Nutrition”, “Enteral Immunonutrition”, “Random” and “Randomized Controlled Trial”. TITLE-ABS-KEY was used for searching Scopus with the same keywords above. In Cochrane database enteral immunonutrition was used as key term. The PICO format was adopted to establish specific selection criteria in which P was referred to the gastric cancer patients undergoing gastrectomy, I was referred to EIN, C was referred to EN, O includes both clinical outcome, immunological and nutrition status index. The design style was limited to randomized controlled trials (RCTs). Only articles published in English language were in criteria.

In this meta-analysis, clinical outcomes included incidence of pulmonary infection, incision infection, mortality, postoperative infectious complications, operating time, SIRS and the LHS. Relevant T cell subsets which included CD4^+^ and CD8^+^. Immune globulin included IgG and IgM. Serum protein which consisted of total protein, albumin, proalbumin and transferring. Lymphocytes was also included.

The following studies were excluded: narrative or expert reviews, non-RCT, experimental data such as animal studies or trials, unable to acquire primary data and essential information from authors, articles published not in English. The following patients were excluded: GC patients combined with other cancers, patients with parenteral nutrition, patients have unresectable neoplasm, immune insufficiency because of endocrine or metabolic disorders, major organic disease, treatment with immunosuppressive drugs, corticosteroids or radiotherapy, severe preoperative infection.

### Quality assessment

Cochrane Collaboration’s tool published in the Cochrane Handbook (version 5.3) was used to evaluate the risk of bias and it contained seven items: random sequence generation, blinding of participants and personnel, allocation concealment, blinding of outcome assessors, selective reporting, incomplete outcome data and other biases. The risk of bias assessment was carried out by two reviewers independently (YC and JFZ). A third reviewer (JW) arbitrated unresolved disagreements. Finally, the potential bias was graded as “high risk” “low risk” or “unclear risk”.

### Statistical analysis

Review Manager (RevMan) 5.3 was used to characterize the effect of various dichotomous and continuous outcomes. Reference management software (Endnote) was used to manage, extract data and delete duplicate references. Forest plots were generated to evaluate the effect of outcome variables for all the studies. Dichotomous outcomes were assessed by relative risk (RR) with 95% confidence interval (CI). Mean difference (MD) with 95% CI was adopted to express the continuous outcome data, if all the studies included with the same unit and magnitude; otherwise, standard mean difference (SMD) was adopted. Heterogeneity was measured through χ^2^ test with corresponding *P* value and I^2^ test [15]. If between-study heterogeneity existed (I^2^ > 50% or *P* < 0.05), random-effects model was used; otherwise, the pooled analysis was done with fixed-effect model. A *p*-value of less than 0.05 was considered as statistically significant. Detection time of indicators of interest was defined into two subgroups (D ≥ 7 and D < 7, post-operatively). If necessary, we removed one or two studies to make the heterogeneity (I^2^) getting close to zero.

## Results

**Fig. 1:**
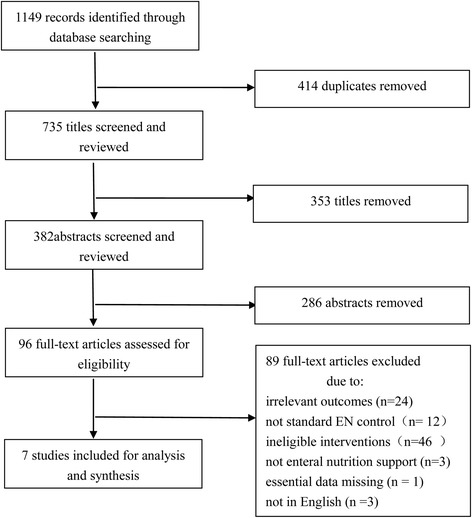
Study selection flow diagram

In this meta-analysis, 1149 unique studies were initially identified across the four electronic databases, after removal of 414 duplicates. 96 studies were eligible to further full-text screening, of which 89 articles did not meet the inclusion criteria, and the rest of 7 studies with 583 subjects were included in the finally analysis. The flow diagram with detailed information was outlined in Fig. 1.

The characteristics of articles included were listed in Table 1. Five out of seven trials were done to compare the EIN with standard EN, one trial was for comparing EIN with oral placebo, and one trial was for comparing EIN with regular diet. About half of articles (*n* = 4, 57%) reported both laboratory indices and clinical indicators, two targeted clinical outcomes only and one restricted the analysis to laboratory indices. Most studies included more than one immunonutrition (Arg, Gln, ω-3-FAs and RNA), with the remainder one study conducted with Gln only. Most studies applied the EIN after surgery, and two administered trial before operation. The sample size of study ranged from 31 [16] to 231 [17]. Patients in most articles aged ≥ 65 years, with only one aged < 60 years [18]. Three of the seven studies were from Japan, two conducted in China, one in Spain and one in Italy.

**Table 1:** Characteristics of 7 eligible studies

Author (year) [Ref]	Country	Diagnosis	Age of patients (Years)	Sample size (EIN/EN)	Elements of EIN	Nature of EN	EIN initiation time	Total during time of nutrition support (days)	Mode of enteral feeding	Reported Outcomes
Liu et al. (2012) [18]	China	Advanced gastric cancer	57.3 ± 7.1 (EIN) 58.4 ± 6.3 (EN)	28/24	Arg and Gln	Standard EN	Post-operation	7	Nasoenteral	Total protein, albumin, proalbumin, transrerrin, CD4+, CD8+, IgM, IgG, LHS, postoperative complications, incision infection, pulmonary infection
Okamoto et al. (2009) [20]	Japan	Gastric carcinoma	66.9 ± 11.5 (EIN) 70.9 ± 13.2(EN)	30/30	Arg, ω-3-FAs and RNA	Standard EN	Pre--operation	7	Oral	CD4+, CD8+, CD4+/CD8+, SIRS, lymphocyte, LHS, postoperative complications, operation time, intraoperative blood loss
Chen et al. (2005) [10]	China	Gastric carcinoma	unclear	20/20	Arg, Gln, and ω-3-FAs	Standard EN	Post-operation	7	Nasoenteral	Proalbumin, albumin, transrerrin, CD4+, CD8+, CD4+/CD8+, IgM, IgG
Mochiki et al. (2011) [16]	Japan	Gastric cancer	65 ± 2.6 (EIN) 59 ± 2.1 (EN)	15/16	Gln	Oral placebo	Post-operation	unclear	Oral	Operation time, intraoperative blood loss
Farreras et al. (2005) [19]	Spain	Gastric cancer	66.7 ± 8.3 (EIN) 69.2 ± 13.8(EN)	30/30	Arg,Gln and ω-3-FAs	Standard EN	Post-operation	7	Oral	Total protein, proalbumin, albumin, lymphocyte, incision infection, pulmonary infection, postoperative complications, mortality
Marano et al. (2013) [21]	Italy	Gastric adenocarcinoma	66.6 (55-78) (EIN) 65.1 (49-83) (EN)	54/55	Arg,Gln, ω-3-FAs and RNA	Standard EN	Post-operation	7	Oral	Total protein, albumin, transrerrin, CD4+, CD8+, lymphocyte, LHS, SIRS, postoperative complications, operation time, incision infection, mortality, intraoperative blood loss
Fujitani et al. (2012) [17]	Japan	Gastric adenocarcinoma	64 (26-78) (EIN) 65(30-79) (EN)	120/111	Arg and RNA	Regular diet	Pre--operation	5	Oral	mortality, pulmonary infection, postoperative complications

### Quality assessment

**Fig. 2:**
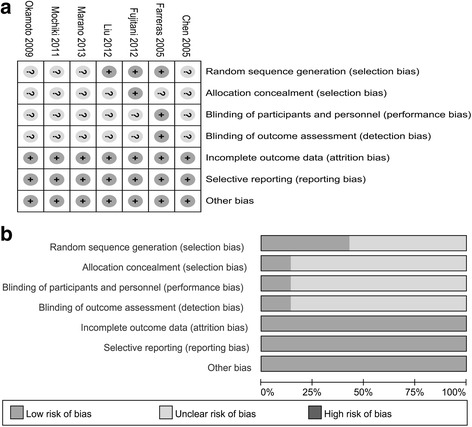
Risk of bias assessment based on review author’s judgement about risk of bias item for each eligible study (*n* = 7). **a** risk of bias summary: : low risk of bias; unclear risk of bias. **b** risk of bias graph presented as percentages across seven studies

Quality assessment of the seven eligible studies are listed in Fig. 2 (a and b). three articles reported methods regarding randomization sequence generation [17–19], only one study [17] performed allocation concealment, only one study [19] performed binding both of participant, personnel and outcome assessment. All the studies reported incomplete outcome data, reporting and other bias. Thus, corresponding domain was assessed as “low risk”, and no other bias sources were assessed in this meta- analysis.

### Meta-analysis on laboratory indices

**Fig. 3:**
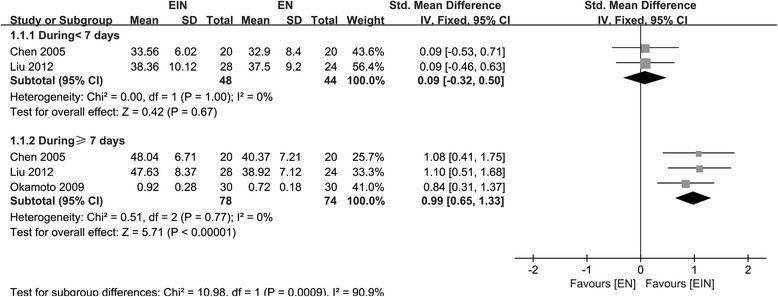
Forest plot on CD4^+^ level comparison between EIN and EN after removal of an article with heterogeneity

**Fig. 4:**
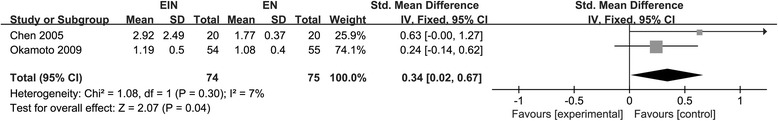
Forest plot on CD4^+^/CD8^+^ comparison between EIN and EN beyond a 7-day time-frame

All the indices were compared between EIN and EN within a 7-day time-frame (D < 7) and beyond a 7-day time-frame post-operatively (D ≥ 7), respectively. One study performed by Yoshiki Okamoto et al. [20], did not report the results of D < 7. CD4^+^ and CD8^+^ indicators were reported in four studies including 261 patients [10, 18, 20, 21]. SMD with 95% CI was used as corresponding effect size because of the different units used across studies. The data of D < 7 and D ≥ 7 were both deemed to be heterogeneity (χ^2^ = 12.74, *P* = 0.002, I^2^ = 84.0%; χ^2^ = 170.69, *P* < 0.00001, I^2^ = 98.0%, respectively), therefore, random-effect model was adopted. The significant difference was not found between the two groups both for D < 7 and D ≥ 7 (SMD = - 0.28; 95% CI, - 0.14–0.47; *P* = 0.46; SMD = - 0.20; 95% CI, - 2.48–2.07; *P* = 0.86, respectively). To find out the source of large heterogeneity, we did a sensitivity analysis and exclude the results conducted by Marano et al. [21] to make the I^2^ to 0.0%. The pooled results were recalculated through a fixed-effect model, and CD4^+^ level had a significant increase on D ≥ 7 in EIN (SMD = 0.99; 95% CI, 0.65–1.33; *P* < 0.00001) (Fig. 3). For CD8^+^, a large heterogeneity was also identified on D < 7 (χ^2^ = 66.98, *P* < 0.00001, I^2^ = 97.0%) and D ≥ 7 (χ^2^ = 116.66, *P* < 0.00001, I^2^ = 97.0%); therefore, random-effect model was used, and we did not find the significant differences both for D < 7 (SMD = - 1.09; 95% CI, - 3.01–0.82; *P* = 0.26) and on D ≥ 7 (SMD = - 0.68; 95% CI, - 2.45–1.09; *P* = 0.45). Two studies including 100 patients [10, 20] reported the CD4^+^/ CD8^+^. It was deemed to be homogeneity (χ^2^ = 1.08, *P* = 0.30, I^2^ = 7.0%), fixed-effect model was adopted. EIN could significantly increase CD4^+^/ CD8^+^ on D ≥ 7 (SMD = 0.34; 95% CI, 0.02–0.67; *P* = 0.04) (Fig. 4).

**Fig. 5:**
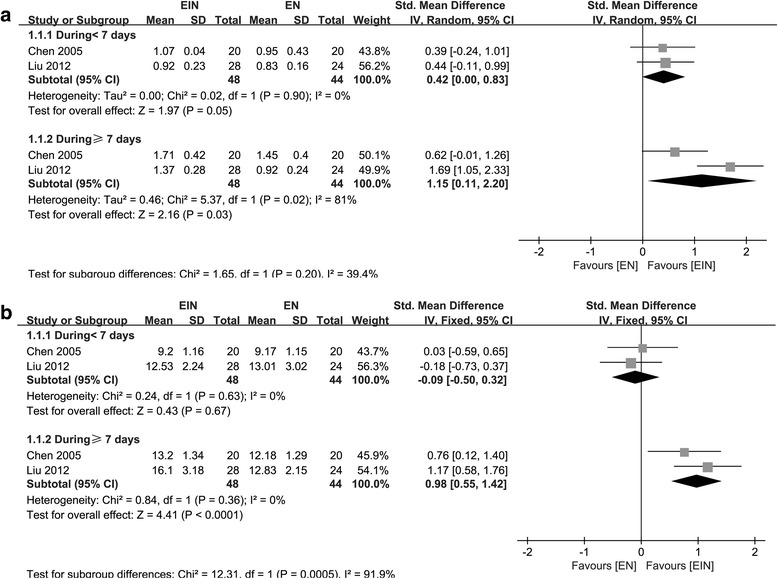
Forest plot on IgM (**a**) and IgG (**b**) comparison between EIN and EN within and beyond a 7-day time-frame

IgM and IgG were measured in two studies including 92 participants [10, 18]. As for IgM, the data on D < 7 was homogeneity (χ^2^ = 0.02, *P* = 0.90 I^2^ = 0.0%), however, statistical heterogeneity was identified from the data on D ≥ 7 (χ^2^ = 5.37, *P* = 0.02, I^2^ = 81.0%); No significant difference was found between two groups on D < 7 (SMD = 0.42; 95% CI, 0.00–0.83; *P* = 0.05), however, IgM was significantly increased in EIN on D ≥ 7 (SMD = 1.15; 95% CI, 0.11–2.20; *P* = 0.03) (Fig.5a). For IgG, the data of D < 7 and D ≥ 7 were both homogeneity (χ^2^ = 0.24, *P* = 0.63, I^2^ = 0.0%; χ^2^ = 0.84, *P* = 0.36, I^2^ = 0.0%, respectively); fixed-effect model was used to perform the analyses. On D < 7, no significant difference was found between the two groups (SMD = - 0.09; 95% CI, - 0.50–0.32; *P* = 0.67), when the time extended to ≥ 7, effect on IgG level appeared (SMD = 0.98; 95% CI, 0.55–1.42; *P* < 0.0001) (Fig. 5b).

**Fig. 6:**
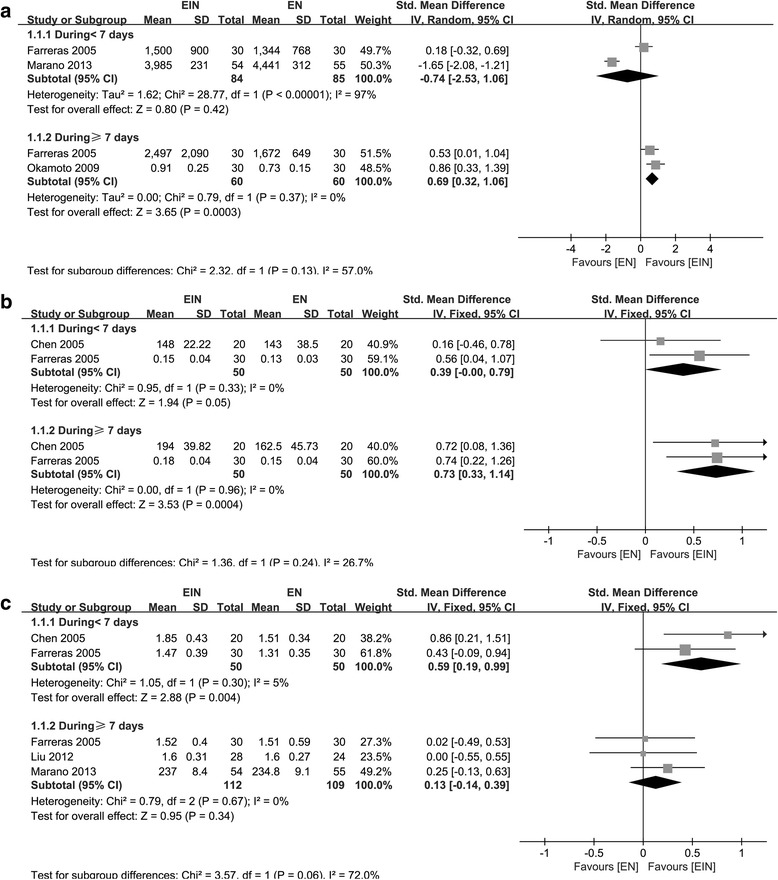
Forest plot on lymphocyte (**a**), proalbumin (**b**) and transferring (**c**) comparison between EIN and EN within and beyond a 7-day time-frame after removal of one or two articles with heterogeneity

Lymphocyte was measured in three studies including 229 patients [19–21]. Yoshiki Okamoto et al. [20] did not report the results on D < 7. A large amount of heterogeneity was observed both on D < 7 and D ≥ 7 (χ^2^ = 28.77, *P* < 0.00001, I^2^ = 97.0%; χ^2^ = 185.51, *P* < 0.00001, I^2^ = 99.0%, respectively); random-effect model was used, and no significant difference was tested between two groups (SMD = - 0.74; 95% CI, - 2.53–1.06; *P* = 0.42; SMD, - 1.54; 95% CI, - 4.99–1.90; *P* = 0.38, respectively). To achieve the relative homogeneity, a study published by Marano et al. [21] was removed (χ^2^ = 0.79, *P* = 0.37, I^2^ = 0.0%). The recalculation results found EIN could significantly increase the lymphocyte level on D ≥ 7 (SMD = 0.69; 95% CI, 0.32–1.06; *P* = 0.0003) (Fig. 6a).

The nutrition status indicators, such as total protein, transferrin, albumin, and proalbumin in serum was measured in three studies including 221 patients [18, 19, 21], four studies including 261 participants, [10, 18, 19, 21], three studies [10, 18, 19] enrolling 152 subjects and four studies recruiting 261 participants [10, 18, 19, 21] respectively. For total protein, heterogeneity existed on D < 7 and D ≥ 7 (χ^2^ = 6.04, *P* = 0.05, I^2^ = 67.0%; χ^2^ = 6.93, *P* = 0.03, I^2^ = 71.0%, respectively); the synthesized results showed no significant differences between the two groups both on D < 7 and D ≥ 7 (SMD = 0.15; 95% CI, - 0.33–0.63; *P* = 0.54; SMD = 0.23; 95% CI, - 0.28–0.75; *P* = 0.37, respectively) through random-effect model. As for albumin, the studies included were homogeneity both on D < 7 and D ≥ 7 (χ^2^ = 0.54, *P* = 0.91, I^2^ = 0.0%; χ^2^ = 1.74, *P* = 0.63, I^2^ = 0.0%, respectively), however, the effect on EIN was also not obvious (SMD = 0.16; 95% CI, - 0.08–0.41; *P* = 0.19 for D < 7; SMD = 0.21; 95% CI, - 0.03–0.46; *P* = 0.08 for D ≥ 7). As for proalbumin, statistical heterogeneity existed from the data on D < 7 and D ≥ 7 (χ^2^ = 3.58, *P* = 0.17, I^2^ = 44.0%; χ^2^ = 7.30, *P* = 0.03, I^2^ = 73.0%, respectively); no significant difference was found between the two groups (SMD = 0.19; 95% CI, - 0.24–0.62; *P* = 0.38; SMD = 0.41; 95% CI, - 0.22–1.04; *P* = 0.20, respectively). However, after excluding the study published by Liu et al. [18], data of proalbumin included deemed to be homogeneity (χ^2^ = 0.00, *P* = 0.96, I^2^ = 0.0%), proalbumin level raised in EIN on D ≥ 7 (SMD = 0.73; 95% CI, 0.33–1.14; *P* = 0.0004) (Fig.6b). As for transferrin, statistical heterogeneity also existed both on D < 7 and D ≥ 7 (χ^2^ = 25,23, *P* < 0.0001, I^2^ = 88.0%; χ^2^ = 6.24, *P* = 0.10, I^2^ = 52.0%, respectively); and no significant difference was found (SMD = 0.07; 95% CI, - 0.67–0.82; *P* = 0.84; SMD = 0.27; 95% CI, - 0.10–0.64; *P* = 0.15, respectively). However, when two studies published by Marano et al. and Liu et al. [18, 21] are excluded, the heterogeneity disappeared (χ^2^ = 1.05, *P* = 0.30, I^2^ = 5.0%), and the effect of EIN on transferring level at D < 7 appeared (SMD = 0.59; 95% CI, 0.19–0.99; *P* = 0.004) (Fig. 6c).

### Synthesis results on clinical outcomes

**Fig. 7:**
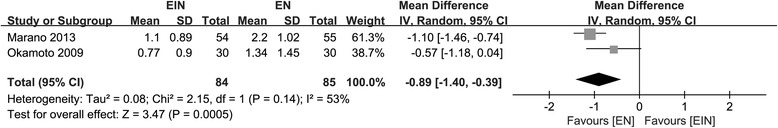
Forest plot on systemic inflammatory response syndrone (SIRS) comparison between EIN and EN

Three studies reported the index regarding length of hospitalization (LHS) [18, 20, 21] which enrolled 221 participants. Statistical heterogeneity was identified (χ^2^ = 13.43, *P* = 0.001, I^2^ = 85.0%); there is no significant difference between two groups (MD = - 1.42; 95% CI, - 4.50–1.66; *P* = 0.37). The data for operating time were also reported in 3 studies [16, 20, 21] which included 200 participants. Heterogeneity was large (χ^2^ = 30.63, *P* < 0.00001, I^2^ = 93.0%) and the pooled-results did not reach the statistical significance between groups (SMD = - 0.43; 95% CI, - 1.65–0.78; *P* = 0.48). The data for systemic inflammatory response syndrone (SIRS) were reported in 2 studies [20, 21] including 169 participants. Statistical heterogeneity was identified, though *P* > 0.05 in χ^2^ test (χ^2^ = 2.15, *P* = 0.14, I^2^ = 53.0%); the meta-analysis was done by random effect model and indicated patients received EIN had less SIRS (MD = - 0.89; 95% CI, - 1.40 to - 0.39; *P* = 0.005) (Fig. 7).

**Fig. 8:**
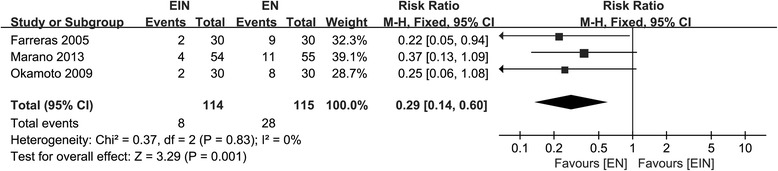
Forest plot on postoperative complications comparison between EIN and EN after removal of two articles with heterogeneity

Pulmonary infection, incision infection, mortality and overall postoperative infectious complications were reported in three studies including 343 patients [17–19], three studies including 221 participants [18, 19, 21], three studies enrolling 300 subjects, [17, 19, 21] and five studies recruiting 512 subjects [17–21] respectively. The meta-analysis on pulmonary infection and postoperative complications indicated statistical heterogeneity; incision infection and mortality were all deemed to be homogeneity, (χ^2^ = 4.17, *P* = 0.12, I^2^ = 52.0% for pulmonary infection; χ^2^ = 11.1, *P* = 0.03, I^2^ = 64.0% for postoperative complications; χ^2^ = 1.67, *P* = 0.43, I^2^ = 0.0% for incision infection; χ^2^ = 0.15, *P* = 0.70, I^2^ = 0.0% for mortality). The synthesized results presented no significant differences between groups regarding these data (RR = 1.02; 95% CI, 0.16–6.50; *P* = 0.98 for pulmonary infection; RR = 0.57; 95% CI, 0.28–1.14; *P* = 0.11 for postoperative complications; RR = 0.52; 95% CI, 0.18–1.53; *P* = 0.24 for incision infection; RR = 0.67; 95% CI, 0.12–3.89; *P* = 0.66 for mortality). However, the heterogeneity reduced to zero by removing two studies conducted by Fujitani et al. and Liu et al. [17, 18], patients in EIN group had lower probability to occur postoperative complications (RR = 0.29; 95% CI, 0.14–0.60; *P* = 0.001) (Fig. 8). however, pulmonary infection was also the same between the two groups.

## Discussion

GC is the fourth most common tumors and the second leading cause of cancer deaths worldwide [22]. Patients with GC often suffer with malnutrition and it will be more severe when tumorectomy was required [17]. Malnutrition is usually related to impaired cellular and humoral immune function, inflammatory response changes, and wound healing process delay or failure [20]. In perioperative patients, nutrition support strategy become a popular and essential way [23, 24]. Nutritional therapy includes PN and EN, the latter one is generally more frequently preferred, because it is safer and having more physiological and economic benefits [25, 26]. EN has been supplied to patients with critical diseases using a variety of nutritional regimens. There has been an increasing recognition that certain essential nutrients can modulate a series of metabolic, inflammatory and immune processes when ingested more than the normal daily requirements. However, the clinical effect was poor than expected due to the complexity of tumor [27]. EIN was an alternative way and proposed to be better therapy to modulate metabolism and immune response. ESPEN (European Society for Clinical Nutrition and Metabolism) has also recommended the use of EIN in surgical patients suffering from upper gastrointestinal cancer to reduce major infectious complications [5]. Although reduction of post-operative complications and some other positive effects of the EIN therapy were reported in some studies [28]. Whether EIN was superior than EN in case of clinical indices (such as hospital stay and postoperative infection) and immune indices was still in dispute.

GM Song et al. in 2015 [13] performed a meta-analysis to assess the influence of EIN for GC patients after surgery both on clinical and immunological outcomes. This meta-analysis showed that EIN could effectively improve the GC patient’s nutritional and immunological status who undergoing surgical resection. It can effectively relieve the inflammatory response and enhance the host immunity. Several immune related factors were increased, such as CD4^+^, CD4^+^/CD8^+^, CD3^+^, IgA, IgG, IgM and NK cells, while some inflammatory related cytokines (e.g. IL-6 and TNF-a) were reduced. However, EIN did not improve the level of CD8^+^ and serum protein. Morbidity of postoperative complications, and the length of hospitalization were not improved either.

Compared with the meta-analysis conducted by GM Song et al. [13], our research was more comprehensive due to more detailed search strategy through each nutrient substance such as Arg, Gln, ω-3-FAs and RNA. Moreover, our study focus on GC patients only and have a more specific application effect regarding EIN comparing with the recently published meta-analysis [12]. We refined the time points of each test index into ≥ 7 and < 7 days post-operatively, rather than the general comparison of preoperative and postoperative. The level of CD4^+^, CD4^+^/ CD8^+^, the IgM, the IgG, the lymphocyte and the proalbumin were effectively increased in EIN on D ≥ 7, but the effect was not obvious on D < 7. It may indicate the time effect of EIN with those indices. The level of CD8^+^ and the serum protein except proalbumin were not improved whatever D < 7 or D ≥ 7. In addition, clinical indicators such as SIRS and overall postoperative infectious complications were also improved. The postoperative infectious complication included respiratory tract infection, urinary tract infection, sepsis, intraabdominal abscess and surgical wound infection. However, single infectious event such as pulmonary infection, incision infection did not change. The improvements of postoperative infectious complication may be primarily manifested in the other part of the body. Nevertheless, the LHS was not shorter in EIN compared to EN. In addition to the postoperative complication, LHS was affected by medical insurance system and the hospital manage mode. The mortality and operating time were not influenced either.

Supply of immunomodulatory nutrients (e.g., ω-3-FAs, Arg and dietary nucleotides) could promote the maintaining of homeostasis postoperatively and reduce inflammatory response [29–32]. Arginine is thought to be a enhancer to T-cells, which could proliferate in response to mitogens or cytokines stimulation [33, 34]. Like those results reported, our meta-analysis confirmed the increase of CD4^+^ belonging to the T cells in EIN group indicating the enhancement of cellular immunity. Meanwhile, the higher concentration of immunoglobulin IgM and IgG may be an indication of inflammatory response relieve and host immunity enhancement [8, 35]. All the improved data suggested that EIN could effectively improve the inflammatory responses and postoperative immune function after gastric surgery by regulating the immune function [20, 36]. Otherwise, lymphocytes and the serum protein, the incidence of pulmonary infection, incision infection and other clinical outcomes cannot be effectively increased by EIN. It can be explained that EIN play little role on those variants.

A major strength of this review is broad search terms with specific immunonutrition element and multiple database. A rigorous screening process was conducted in data searching, extraction and quality appraisal by two researchers independently. Even so, there are some limitations need to be demonstrated. First, exclusion of grey literature and non-English language studies was the key limitation in this study. Second, publish biaswere not conducted because of the small number of included articles and it may incline to no statistical significance. Finally, some other indicators related with EIN effect were not mentioned due to the incomplete data.

## Conclusion

This synthesis analyses clearly show that EIN is better to EN in improving the immune function for patients with gastric cancer after surgery. Although the incidence of pulmonary infection, LHS and other clinical outcomes were not improved, EIN is clinically feasible and safe to be recommended as nutritional support in major gastric surgery.

## Abbreviations

Arg: Arginine
CI: Confidence interval
EIN: Enteral immunonutrition
EN: Enteral nutrition
GC: Gastric cancer
Gln: Glutamine
LHS: Length of hospitalization
MD: Mean difference
PN: Parenteral nutrition
RCTs: Randomized controlled trials
RNA: Ribonucleic acid
RR: Relative risk
SIRS: Systemic inflammatory response syndrone
SMD: Standard mean difference
ω-3-FAs: Omega-3 fatty acids
